# Cognitive impairment and associated factors among community-dwelling older adults with hypertension in tropical China

**DOI:** 10.3389/fpubh.2026.1900335

**Published:** 2026-08-13

**Authors:** Liyu Peng, Xiuru Wang, Wenting Cao, Ping Zhang, Dee Yu, Huajiang Liu, Yong You, Binwen Huang, Jindong Ding Petersen

**Affiliations:** 1 School of Public Health, Key Laboratory of Tropical Translational Medicine of Ministry of Education, Hainan Medical University, Haikou, China; 2 The First Affiliated Hospital of Hainan Medical University, Haikou, China; 3 Department of Neurology, The Second Affiliated Hospital of Hainan Medical University, Haikou, China; 4 School of Intelligent Medicine and Technology, Big Data Research Center, Hainan Engineering Research Center for Health Big Data, Hainan Medical University, Haikou, China; 5 Research Unit of General Practice, Department of Public Health, University of Southern Denmark, Odense, Denmark; 6 Research Unit of General Practice, Department of Public Health, University of Copenhagen, Copenhagen, Denmark

**Keywords:** cognitive impairment, dementia risk, hypertension, older adults, tropical region

## Abstract

**Background:**

Cognitive impairment (CI) is highly prevalent among older adults with hypertension and imposes substantial burdens on individuals, families, and healthcare systems. Identifying factors associated with CI is essential for developing targeted prevention strategies, yet evidence from tropical regions of China remains limited.

**Objective:**

To examine the prevalence of CI and factors associated with CI among community-dwelling older adults with hypertension in tropical China.

**Methods:**

This community-based cross-sectional study included 735 older adults aged ≥60 years with self-reported, physician-diagnosed hypertension recruited from community health centers in Haikou, China, between 2023 and 2025. Cognitive function was assessed using the Chinese Mini-Mental Status with education-adjusted cutoff values. Multivariable logistic regression was performed to identify factors associated with CI. Age- and sex-stratified analyses, interaction analyses, and sensitivity analyses were conducted to evaluate the robustness of the findings.

**Results:**

Among the 735 participants (mean age, 72.3 ± 7.7 years; 57.6% women), 223 (30.3%) had CI. In the fully adjusted model, participants aged ≥80 years had significantly higher odds of CI than those aged 60–69 years (OR = 2.21, 95% CI: 1.34–3.64). Depressive symptoms were also associated with increased odds of CI (OR = 1.87, 95% CI: 1.03–3.39), whereas higher educational attainment, medium monthly income, and higher BMI were associated with lower odds of CI. Stratified analyses suggested potential sex differences in the association between marital status and CI, and formal interaction analysis demonstrated a significant interaction between sex and marital status (P for interaction = 0.015). Sensitivity analyses generally supported the robustness of the primary findings.

**Conclusion:**

CI was common among community-dwelling older adults with hypertension in tropical China. Older age and depressive symptoms were associated with higher odds of CI, whereas higher educational attainment, moderate income, and higher BMI were associated with lower odds. The observed sex differences in the association between marital status and CI highlight the importance of considering social determinants and sex-specific approaches when developing community-based cognitive health strategies for older adults with hypertension.

## 1 Introduction

Cognitive impairment (CI) is characterized by a decline in one or more cognitive domains, such as memory, executive function, language, attention, reasoning, and visuospatial abilities (1). It encompasses a spectrum ranging from mild cognitive impairment (MCI) to dementia, is highly prevalent among older adults, and adversely affects daily living and social functioning, thereby increasing the socioeconomic burden (2). With rapid global population aging, the burden of CI is expected to rise substantially. Worldwide, more than 55 million people were living with dementia globally in 2021, and this number is projected to reach 139 million by 2050 (3). In China, the estimated prevalence of dementia and MCI among adults aged ≥60 years is 6.0 and 15.5%, respectively (4), With the rapidly aging population, the number of people with dementia in China is expected to increase significantly, reaching about 49 million by 2050 (5).

Hypertension is one of the most important vascular risk factors for cognitive decline. Long-standing elevated blood pressure can promote cerebral arteriosclerosis, reduce vascular elasticity, impair cerebral autoregulation, and compromise cerebral perfusion. These changes may increase susceptibility to recurrent cerebral ischemia, white matter damage, and potentially lead to progressive cognitive decline **(****6****)**.

The public health relevance of hypertension is particularly substantial in China, where approximately 245 million people have hypertension, and the prevalence exceeds 55% among adults aged ≥65 years (7). Globally, the economic burden of hypertension accounts for approximately 10% of worldwide healthcare expenditures (8). A recent meta-analysis reported that the prevalence of CI among people with hypertension in China was approximately 37.6%, indicating a considerable disease burden in this high-risk population (9).

CI among older adults with hypertension is shaped by multiple biological, behavioral, psychological, and social factors. Advanced age may amplify the cerebrovascular effects of hypertension through reduced physiological reserve and impaired recovery from cellular stress (10). Depression, physical inactivity, smoking, diabetes, obesity, and excessive alcohol consumption, and low educational attainment are recognized as potentially modifiable risk factors for dementia (11), and many of these factors also overlap with determinants of hypertension (12–14).

Although CI in older adults with hypertension has been widely studied, evidence from the tropical region of China remains limited. Regional characteristics, including lifestyle, dietary habits, and population structure, may influence both hypertension management and CI. Hainan Province, China’s only tropical island province, has distinct environmental and demographic characteristics; however, community-based evidence on CI among older adults with hypertension in this region is scarce.

Therefore, this community-based cross-sectional study aimed to investigate the prevalence of CI and identify associated sociodemographic, socioeconomic, lifestyle, and clinical factors among older adults with hypertension in Haikou, China. The findings could inform region-specific strategies for cognitive health promotion and integrated hypertension management in tropical China.

## 2 Methods

### 2.1 Study design and participants

This community-based, cross-sectional study was conducted among older adults in the Haikou communities in China between April 1, 2023, and July 1, 2025. Sixteen community health service centers and community health service stations were selected as the study sites. Participants were recruited through convenience sampling at 16 community health service centers and stations, where eligible older adults attending routine health examinations or community health services were invited to participate.

Eligible participants were adults aged ≥60 years who: (1) had resided in Hainan for at least 6 months before data collection; (2) had self-reported physician-diagnosed hypertension; (3) provided written informed consent. Exclusion criteria included individuals with (1) the presence of life-threatening severe illnesses, such as advanced cancer or severe cardiac disease; (2) a diagnosis of severe mental disorders, including schizophrenia, schizoaffective disorder, or epilepsy-related psychiatric disorders; (3) severe visual or hearing impairment that interfered with questionnaire completion.

Based on previous literature, the prevalence of CI among Chinese adults aged ≥60 years was estimated to be 22.2% (15). The required sample size was calculated using the standard formula for cross-sectional studies:

$$N=\frac{Z_{1-\alpha /2}^{2}\times p(1-p)}{d^{2}}$$

where *α* was set at 0.05, $Z_{1-\alpha /2}\;$ = 1.96, the estimated prevalence (p) was 22.2%, and the allowable error (d) was defined as 15% of the estimated prevalence (d = 0.15p). The minimum required sample size was calculated to be 598 participants. To account for potential non-response and incomplete data, the calculated sample size was increased by 20%, resulting in a final minimum sample size of 718 participants.

The study was approved by the Ethics Committee of Hainan Medical University (No. HYLL-2022-301). All participants provided written informed consent for research participation.

### 2.2 Covariates and measurements

Data were collected via face-to-face interviews conducted by trained staff using structured questionnaires.

Sociodemographic variables included age, sex, educational attainment, marital status, and personal monthly income. Educational attainment was categorized as illiterate (<1 year), primary school (1–6 years), and junior high school or above (≥7 years). Marital status was classified as married, unmarried, widowed, or divorced. Personal monthly income was grouped as low (<2000 yuan), medium (2000 to 4,999 yuan), or high (≥5,000 yuan).

Height and weight were measured with a combined height-weight stadiometer. Participants were instructed to stand barefoot and without headwear and to stand upright facing forward for the measurement.

Smoking status, alcohol consumption, and daily outdoor activity duration were assessed by self-report. Participants were asked whether they are smokers (Yes/No). Alcohol consumption was evaluated using the question, “In the past 12 months, how often have you consumed any alcoholic beverage?” with responses categorized as daily, 5–6 days/week, 3–4 days/week, 1–2 days/week, 1–3 days/month, or never. Participants who reported alcohol consumption at any frequency ranging from daily to 1–3 days/month were classified as current drinkers; those responding “never” were classified as non-drinkers. Daily outdoor activity duration was categorized as <1, 1–2, 2–4, or ≥4 h/day.

Information on chronic health conditions was obtained via self-report. Participants were asked whether they had ever received a physician’s diagnosis of diabetes, osteoarthritis, heart disease, or hyperlipidemia (Yes/No).

Depressive symptoms were assessed using the 2-item Patient Health Questionnaire (16), with scores ranging from 0 to 6, and a score of 3 or greater indicating clinically significant depressive symptoms.

### 2.3 Cognitive function and measurement

The cognitive function of the older adults participants was assessed using the Chinese Mini-Mental Status (CMMS) (17). The CMMS assesses five cognitive domains: orientation, registration, attention and calculation, recall, and language and visuospatial abilities, with a total score of 30 points; higher scores indicate better cognitive function. The criteria for CI were adjusted according to educational attainment: scores of ≤17 for illiterate participants, ≤20 for those with a primary education, and ≤24 for those with junior high school or above were used to define CI. Scores exceeding these thresholds were considered indicative of normal cognitive function (18).

To assess the robustness of the screening results, an alternative set of education-adjusted cutoff scores for the CMMS was applied to define CI. CI was defined using education-adjusted cutoff scores of the CMMS. Participants were classified as having CI if their CMMS scores were ≤16 for illiterate individuals (formal education <1 year), ≤19 for those with primary school education (1–6 years), or ≤23 for those with middle school education or above (≥7 years) (18).

### 2.4 Data collection and quality control

To ensure consistency in the survey process and reliability of the collected data, all investigators received standard training. Cross verification of the recordings was performed on the same day that the questionnaires were completed. At the end of each survey day, project team members reviewed the audio recordings. They cross-checked against the electronic questionnaire entries to promptly identify and correct omissions or errors, to ensure data quality.

### 2.5 Statistical analysis

Statistical analyses were performed using SPSS version 20.0 (IBM Corp., Armonk, NY, USA). Complete-case analysis was performed because the proportion of missing data was low. Categorical variables are presented as frequencies and percentages and were compared using the χ^2^ test. Continuous variables are presented as means ± standard deviations (SDs) and were compared using independent-samples t tests. Univariate and multivariable logistic regression analyses were performed to examine factors associated with CI. Variables included in the multivariable logistic regression models were selected *a priori* based on previous literature, clinical relevance, and potential confounding rather than solely on statistical significance in univariate analyses. Odds ratios (ORs) and 95% confidence intervals (CIs) were calculated. A two-sided *p* value <0.05 was considered statistically significant. Sensitivity analyses were conducted using an alternative education-adjusted definition of CI to evaluate the robustness of the primary findings. Subgroup analyses were performed according to age and sex. Multiplicative interaction terms between sex and marital status and between sex and educational attainment were entered separately into the fully adjusted logistic regression models, and statistical significance of interaction was assessed using Wald tests.

## 3 Results

### 3.1 Characteristics of the study participants

A total of 735 community-dwelling older adults with hypertension were included in the present study; the mean age of the participants was 72.3 ± 7.7 years, and 57.6% were women. Participants aged 70–79 years accounted for the largest proportion of the study population (41.8%), while 17.3% were aged ≥80 years. Most participants had attained junior high school or above (70.9%) and were married or cohabiting with a spouse (80.8%). In addition, 43.9% reported a medium monthly income of 2000–4,999 yuan. The majority of participants were non-smokers (88.9%) and non-drinkers (83.1%). Baseline characteristics of the study population are summarized in Table 1, and the complete univariate logistic regression results are presented in Supplementary Table S1.

**Table 1:** Sociodemographic and lifestyle characteristics of older adults with hypertension.

Characteristic N (%)	Overall 735 (100)	Normal 512 (69.7)	CI 223 (30.3)	*p*
Age, mean (SD), y	72.3 (7.7)	71.4 (7.2)	74.4 (8.5)	< 0.001
Age groups, y				< 0.001
60–69	301 (40.9)	228 (44.5)	73 (32.7)	
70–79	307 (41.8)	217 (42.4)	90 (40.4)	
≥80	127 (17.3)	67 (13.1)	60 (26.9)	
Sex				0.007
Male	312 (42.4)	234 (45.7)	78 (35.0)	
Female	423 (57.6)	278 (54.3)	145 (65.0)	
Marital status				0.006
Married	590 (80.3)	424 (82.8)	166 (74.4)	
Other status	140 (19.0)	84 (16.4)	56 (25.1)	
Missing	5 (0.7)	4 (0.8)	1 (0.4)	
Educational attainment, y				< 0.001
<1	77 (10.5)	30 (5.9)	47 (21.1)	
1–6	137 (18.6)	98 (19.1)	39 (17.5)	
≥7	521 (70.9)	384 (75.0)	137 (61.4)	
Monthly income, yuan				< 0.001
Low <2000	226 (30.7)	133 (26.0)	93 (41.7)	
Medium 2000 to <5,000	307 (41.8)	232 (45.3)	75 (33.6)	
High ≥5,000	166 (22.6)	124 (24.2)	42 (18.9)	
Missing	36 (4.9)	23 (4.5)	13 (5.8)	
Smoking status				0.849
Yes	81 (11.0)	57 (11.1)	24 (10.8)	
No	649 (88.3)	450 (87.9)	199 (89.2)	
Missing	5 (0.7)	5 (1.0)	0 (0)	
Alcohol consumption				0.037
Yes	124 (16.9)	96 (18.7)	28 (12.6)	
No	609 (82.8)	414 (80.9)	195 (87.4)	
Missing	2 (0.3)	2 (0.4)	0 (0)	
Outdoor activity duration, h/day				0.491
<1	249 (33.9)	175 (34.2)	74 (33.2)	
1 - < 2	295 (40.1)	206 (40.2)	89 (39.9)	
2 - < 4	124 (16.9)	90 (17.6)	34 (15.3)	
≥4	54 (7.3)	33 (6.4)	21 (9.4)	
Missing	13 (1.8)	8 (1.6)	5 (2.2)	
BMI, mean (SD), kg/m^2^	24.4 (3.4)	24.7 (3.3)	23.9 (3.5)	0.004
Chronic disease (Yes)				
Diabetes mellitus	179 (24.5)	120 (23.6)	59 (26.5)	0.412
Heart disease	135 (18.6)	101 (20.0)	34 (15.3)	0.132
Hyperlipidemia	190 (26.1)	138 (27.3)	52 (23.3)	0.262
Osteoarthritis	194 (26.4)	140 (27.3)	54 (24.2)	0.376
Depressive symptoms	61 (8.4)	33 (6.5)	28 (12.7)	0.005

CI, cognitive impairment, defined according to education-adjusted CMMS cut-off scores (≤17 for illiterate participants, ≤20 for primary school education, and ≤24 for junior high school education or above). BMI, body mass index; other status: unmarried, widowed, or divorced.

CMMS identified 223 participants (30.3%) having CI according to education-adjusted criteria.

Participants with CI were generally older, had lower educational attainment, lower income, lower BMI, and a higher prevalence of depressive symptoms than those without CI. In contrast, smoking status, outdoor activity duration, diabetes, heart disease, hyperlipidemia, and osteoarthritis were not significantly associated with CI.

### 3.2 Factors associated with cognitive impairment

Table 2 presents the results of the logistic regression analyses examining factors associated with CI among these older adults with hypertension. In the multivariable-adjusted model, advanced age remained significantly associated with increased odds of CI. Compared with participants aged 60–69 years, those aged ≥80 years had more than twofold higher odds of CI (*OR* = 2.21, 95% *CI*: 1.34–3.64).

**Table 2:** Logistic regression analyses of factors associated with cognitive impairment among older adults with hypertension.

Characteristic	Crude model		Adjusted model^a^	
	*OR* (95% *CI*)	*p*	*OR* (95% *CI*)	*p*
Age groups, y				
60–69	1 [Reference]		1 [Reference]	
70–79	1.30 (0.90–1.86)	0.159	1.17 (0.78–1.75)	0.448
≥80	2.78 (1.81–4.33)	<0.001	2.21 (1.34–3.64)	0.002
Sex				
Female	1 [Reference]		1 [Reference]	
Male	0.64 (0.46–0.86)	0.007	0.79 (0.53–1.18)	0.250
Marital status				
Married	1 [Reference]		1 [Reference]	
Other status	1.70 (1.16–2.50)	0.006	1.08 (0.69–1.68)	0.746
Educational attainment, y				
<1	1 [Reference]		1 [Reference]	
1–6	0.25 (0.14–0.46)	<0.001	0.27 (0.14–0.52)	<0.001
≥7	0.23 (0.14–0.38)	<0.001	0.41 (0.23–0.74)	0.003
Monthly income, yuan				
Low <2000	1 [Reference]		1 [Reference]	
Medium 2000 to <5,000	0.46 (0.32–0.67)	<0.001	0.53 (0.35–0.81)	0.003
High ≥5,000	0.48 (0.31–0.75)	0.001	0.65 (0.40–1.08)	0.094
Alcohol consumption				
No	1 [Reference]		1 [Reference]	
Yes	0.62 (0.39–0.98)	0.039	0.82 (0.48–1.41)	0.473
BMI, per 1 kg/m^2^	0.93 (0.89–0.98)	0.003	0.95 (0.90–1.00)	0.041
Depressive symptoms				
No	1 [Reference]		1 [Reference]	
Yes	2.10 (1.23–3.56)	0.006	1.87 (1.03–3.39)	0.038

CI, cognitive impairment, defined according to education-adjusted CMMS cut-off scores (≤17 for illiterate participants, ≤20 for primary school education, and ≤24 for junior high school education or above). OR, odds ratio; 95%CI, 95% confidence interval.

a Adjusted for age, sex, marital status, educational attainment, monthly income, alcohol consumption, BMI, and depressive symptoms.

Educational attainment emerged as one of the strongest factors associated with CI. Compared with illiterate participants, those with primary school education had substantially lower odds of CI (*OR* = 0.27, 95% *CI*: 0.14–0.52), and similar associations were observed among participants with junior high school or above (*OR* = 0.41, 95% *CI*: 0.23–0.74).

Socioeconomic status was also associated with cognitive function. Participants with medium monthly income had significantly lower odds of CI than those with low income (*OR* = 0.53, 95% *CI*: 0.35–0.81). In addition, each 1-unit increase in BMI was associated with 5% lower odds of CI (*OR* = 0.95, 95% *CI*: 0.90–1.00). Depressive symptoms were also associated with higher odds of CI (OR = 1.87, 95% CI: 1.03–3.39). By contrast, sex, marital status, and alcohol consumption were not significantly associated with CI after multivariable adjustment.

### 3.3 Stratified analyses

**Figure 1:**
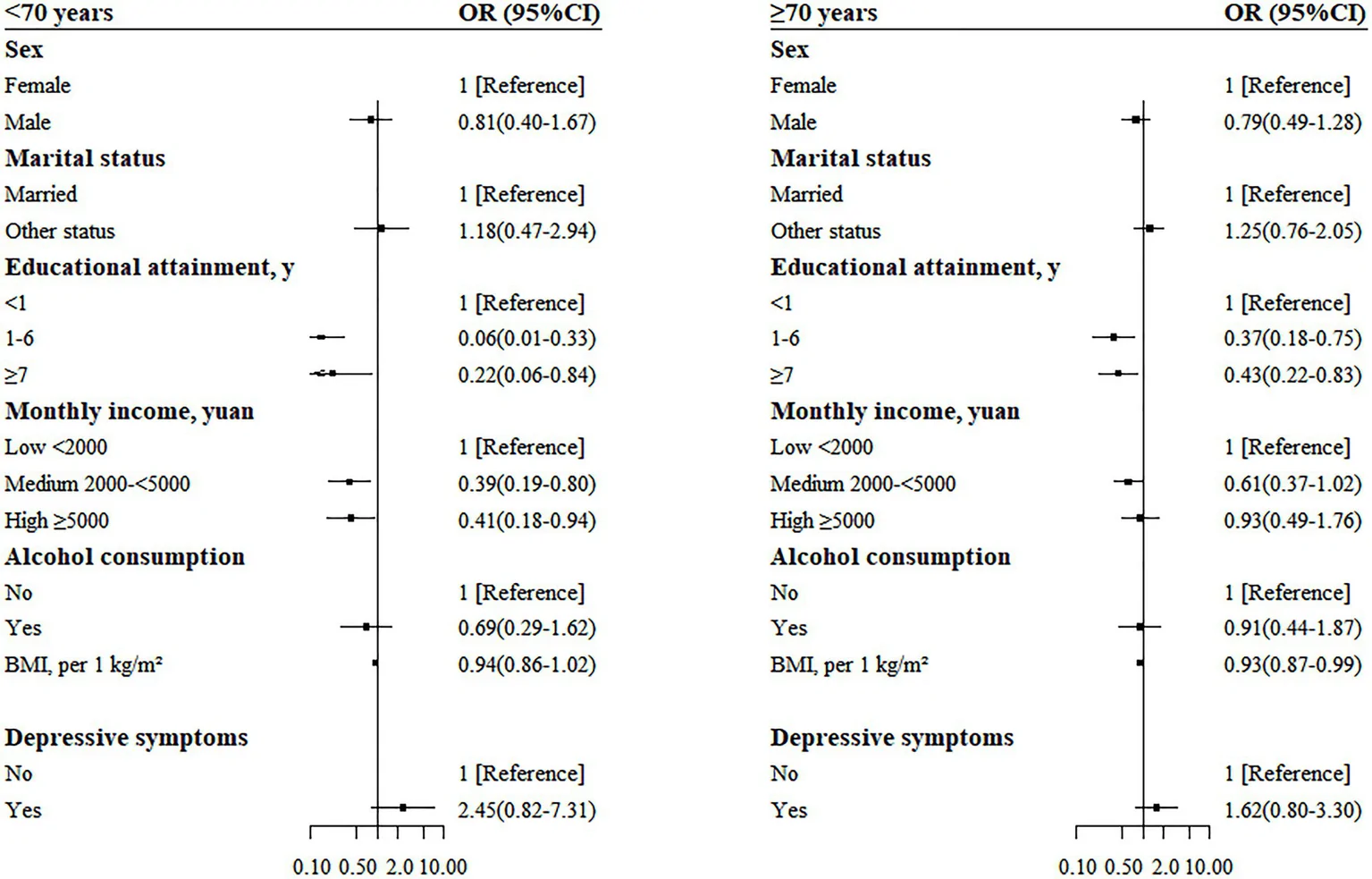
Forest plot showing age-stratified multivariable logistic regression analyses for factors associated with cognitive impairment. Models were adjusted for sex, marital status, educational attainment, monthly income, alcohol consumption, BMI, and depressive symptoms. OR, odds ratio; CI, confidence interval.

Age-stratified analyses showed generally consistent associations between educational attainment and CI across age groups (Figure 1).

Among participants aged <70 years, both primary education (*OR* = 0.06, 95% *CI*: 0.01–0.33) and junior high school or above (*OR* = 0.22, 95% *CI*: 0.06–0.84) were associated with lower odds of CI compared with illiteracy. Similarly, medium and high-income levels were associated with lower odds of CI in this age group. Among participants aged ≥70 years, higher educational attainment remained significantly associated with lower odds of CI. Furthermore, higher BMI was inversely associated with CI, with each 1-unit increase in BMI associated with 7% lower odds of CI (*OR* = 0.93, 95% *CI*: 0.87–0.99). No statistically significant associations were observed for sex, marital status, alcohol consumption, or depressive symptoms in either age subgroup.

**Figure 2:**
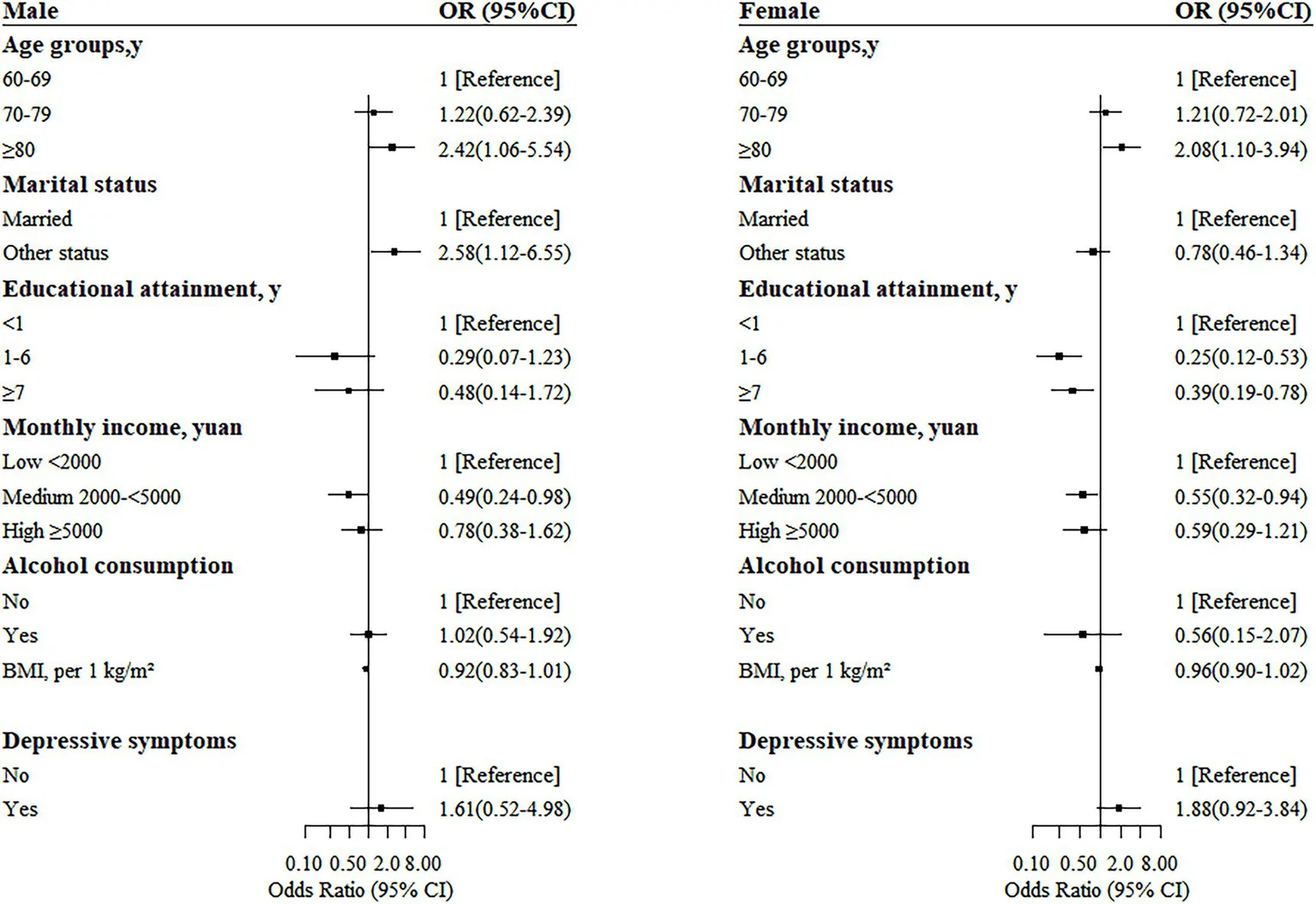
Forest plot showing sex-stratified multivariable logistic regression analyses for factors associated with cognitive impairment. Models were adjusted for age, marital status, educational attainment, monthly income, alcohol consumption, BMI, and depressive symptoms. OR, odds ratio; CI, confidence interval.

Sex-stratified analyses suggested potentially different patterns of factors associated with CI between male and female participants (Figure 2).

Among men, participants aged ≥80 years had significantly higher odds of CI (*OR =* 2.42, 95% *CI*: 1.06–5.54). In addition, unmarried, widowed, or divorced men had higher odds of CI than married men (*OR* = 2.58, 95% *CI*: 1.12–6.55). A medium income level was associated with lower odds of CI among men.

Among women, advanced age was also significantly associated with CI (*OR* = 2.08, 95% *CI*: 1.10–3.94). In contrast to male participants, higher educational attainment was significantly associated with lower odds of CI among women, including both primary education (*OR* = 0.25, 95% *CI*: 0.12–0.53) and junior high school or above (*OR =* 0.39, 95% *CI*: 0.19–0.78). Medium income remained inversely associated with CI in women.

A statistically significant interaction was observed between sex and marital status (P for interaction = 0.015), suggesting that the association between marital status and CI differed significantly between men and women. In contrast, no statistically significant interaction was observed between sex and educational attainment (P for interaction = 0.974), indicating that the association between educational attainment and CI was similar between men and women (Supplementary Table S2).

### 3.4 Sensitivity analysis

To evaluate the robustness of the primary findings, sensitivity analyses were conducted using alternative education-adjusted CMMS cutoff criteria (Table 3). The sensitivity analysis generally supported the robustness of the primary findings,

**Table 3:** Sensitivity analysis using alternative criteria for cognitive impairment.

Characteristic	Crude model		Adjusted model^a^	
	*OR* (95% *CI*)	*p*	*OR* (95% *CI*)	*p*
Age groups, y				
60–69	1 [Reference]		1 [Reference]	
70–79	1.18 (0.79–1.77)	0.420	1.16 (0.74–1.81)	0.516
≥80	2.26 (1.41–3.63)	0.001	1.74 (1.01–2.99)	0.047
Sex				
Female	1 [Reference]		1 [Reference]	
Male	0.66 (0.46–0.95)	0.024	0.79 (0.51–1.23)	0.299
Marital status				
Married	1 [Reference]		1 [Reference]	
Other status	1.66 (1.09–2.51)	0.017	1.10 (0.68–1.77)	0.693
Educational attainment, y				
<1	1 [Reference]		1 [Reference]	
1–6	0.62 (0.34–1.15)	0.130	0.62 (0.32–1.23)	0.173
≥7	0.47 (0.28–0.78)	0.004	0.81 (0.43–1.53)	0.518
Monthly income, yuan				
Low <2000	1 [Reference]		1 [Reference]	
Medium 2000 to <5,000	0.45 (0.30–0.69)	<0.001	0.47 (0.30–0.75)	0.001
High ≥5,000	0.53 (0.33–0.87)	0.011	0.652 (0.38–1.12)	0.123
Alcohol consumption				
No	1 [Reference]		1 [Reference]	
Yes	0.61 (0.36–1.02)	0.061	0.70 (0.37–1.33)	0.275
BMI, mean (SD), kg/m^2^	0.91 (0.86–0.96)	0.001	0.94 (0.88–1.00)	0.020
Depressive symptoms				
No	1 [Reference]		1 [Reference]	
Yes	2.05 (1.17–3.59)	0.012	1.74 (0.94–3.23)	0.079

Cognitive impairment, defined according to education-adjusted CMMS cut-off scores (≤16 for illiterate participants, ≤19 for primary school education, and ≤23 for junior high school education or above). 95% CI, 95% Confidence interval.

a Adjusted for age, sex, marital status, educational attainment, monthly income, alcohol consumption, BMI, and depressive symptoms.

Advanced age remained significantly associated with higher odds of CI, with participants aged ≥80 years showing increased odds of CI compared with those aged 60–69 years (OR = 1.74, 95% CI: 1.01–2.99). Medium income level and higher BMI also remained significantly associated with lower odds of CI.

However, the associations between educational attainment and CI observed in the primary analyses were attenuated and no longer statistically significant after multivariable adjustment. Similarly, depressive symptoms were no longer significantly associated with CI in the sensitivity model, although the direction of the association remained consistent with the primary analysis. Overall, these findings indicate that the associations for age, income, and BMI were relatively stable across different CMMS cutoff criteria.

## 4 Discussion

In this community-based cross-sectional study of older adults with hypertension in a tropical region of China, the prevalence of CI was 30.3%. Advanced age and depressive symptoms were associated with higher odds of CI, whereas higher educational attainment, medium income level, and higher BMI were associated with lower odds of CI. In addition, sex-stratified analyses suggested potential sex differences in the association between marital status and cognitive function.

The prevalence of CI observed in the present study was higher than that reported in the general older Chinese population. Previous nationwide studies in China have reported CI prevalence rates of approximately 22.2% among community-dwelling older adults (15). The relatively higher prevalence in our study may be partly attributable to the inclusion of older adults with hypertension, a well-established vascular risk factor for cognitive decline and dementia (19). Hypertension may contribute to cerebral small vessel disease, endothelial dysfunction, chronic cerebral hypoperfusion, and white matter injury, all of which are associated with impaired cognitive function (20).

However, the prevalence observed in our study was slightly lower than that reported among older adults with hypertension in economically underdeveloped rural regions of Northwest China (21). Regional differences in socioeconomic conditions and environmental exposures may partly explain variations in CI across studies. Lower educational attainment and socioeconomic status have been consistently associated with poorer cognitive outcomes in older populations (22). Additionally, Hainan Province has relatively favorable air quality compared with northern regions of China, and previous studies have suggested that long-term exposure to air pollution is associated with worse cognitive outcomes, while reductions in particulate matter may be associated with slower cognitive decline (23). Nevertheless, compared with economically developed urban regions such as Beijing (24), the prevalence in the present study remained relatively high. This may reflect the combined influence of socioeconomic characteristics, educational levels, and regional contextual factors specific to tropical areas. Although emerging evidence suggests that environmental conditions such as temperature and humidity may be associated with physiological processes relevant to brain health, including sleep regulation and cerebrovascular function (25, 26), these mechanisms remain to be further clarified.

In this study population, advanced age was associated with higher odds of CI, whereas higher educational attainment, moderate monthly income, and higher BMI were associated with lower odds. These findings are consistent with established patterns of association between sociodemographic and vascular factors and cognitive function reported in previous studies (4, 27–30). Depressive symptoms were also associated with CI. This finding is consistent with previous longitudinal and cross-sectional studies reporting associations between depressive symptoms and cognitive decline among older adults with hypertension (31, 32). Although the association between depressive symptoms and CI became attenuated in sensitivity and subgroup analyses, its direction remained generally consistent. Given the high prevalence of both hypertension and depressive symptoms among older adults, incorporating mental health screening into community-based hypertension management may facilitate earlier identification of individuals at increased risk of CI.

Our findings are also broadly consistent with evidence from other tropical and subtropical regions, although both similarities and regional variations in effect magnitude were observed. In Latin America, population-based studies from São Paulo, Brazil, have identified older age, lower educational attainment, and socioeconomic disadvantage as important correlates of CI among community-dwelling older adults, while longitudinal evidence from the Brazilian Health. Well-being and Aging (SABE) study has further shown that socioeconomic disadvantages and poor nutritional status are associated with accelerated cognitive decline over time (33, 34).

In Southeast Asia, similar patterns have been observed. The Epidemiology of Dementia in Singapore study reported a high prevalence of CI among older adults, with age being the most consistent risk factor across ethnic groups, while educational attainment and socioeconomic conditions played a significant protective role (35). Likewise, nationwide evidence from Thailand has identified older age, lower education, and lack of spousal support as important determinants of CI, and depressive symptoms have also been shown to be independently associated with cognitive dysfunction in hypertensive populations in this region (36, 37). Overall, these findings suggest a general consistency in the distribution of key correlates of CI across tropical and subtropical populations, particularly with respect to aging, educational attainment, and socioeconomic status. However, heterogeneity in reported prevalence may reflect differences in study design, cognitive assessment tools, healthcare accessibility, and underlying population structure, including ethnic composition and cardiovascular risk profiles.

Interestingly, higher BMI was associated with lower odds of CI in the present study. Similar findings have been reported in previous studies involving older adults with hypertension and metabolic disorders (38–40). This may be partly explained by the so-called “obesity paradox,” in which higher BMI in older adults may reflect greater nutritional and metabolic reserves that provide resilience against age-related frailty (41). However, evidence regarding the association between BMI and cognitive function remains inconsistent. Some studies have reported adverse associations between obesity and cognitive outcomes (42, 43), while others have suggested non-linear or U-shaped relationships (44). These discrepancies may be related to differences in population characteristics, age distribution, metabolic status, and limitations of BMI as a proxy for body composition. Given the cross-sectional design, causal relationships cannot be inferred, and longitudinal studies are needed to clarify the role of body composition in cognitive aging. Moreover, the magnitude of the observed association between BMI and CI was relatively modest, suggesting that BMI is unlikely to be an independent determinant of CI. Rather, these findings support the multifactorial nature of CI and should be interpreted cautiously pending confirmation in prospective studies.

The significant interaction between sex and marital status suggests that the association between marital status and CI differs by sex. The observed association between being unmarried, divorced, or widowed and higher odds of CI appeared more pronounced in men than in women (45, 46). This finding is consistent with previous research indicating that married older men tend to show better cognitive outcomes than their unmarried counterparts (47). One plausible explanation is the differential availability and utilization of social support networks between men and women. Women generally maintain broader and more diverse social networks and may be more resilient in sustaining social engagement after spousal loss, whereas men may experience more substantial reductions in social support following widowhood, potentially increasing their vulnerability to cognitive decline (48). However, given the cross-sectional design and potential residual confounding (including baseline cognitive status and unmeasured genetic susceptibility such as APOE ε4), these sex-specific patterns should be interpreted with caution.

While a statistically significant interaction was observed for marital status, no evidence of effect modification by sex was found for educational attainment. These findings suggest that sex-specific heterogeneity may not be a uniform feature across different socioeconomic determinants of CI.

Several vascular comorbidities, including diabetes, dyslipidemia, and heart disease, were not significantly associated with CI in the present study. Although numerous studies have reported positive associations between these vascular conditions and cognitive decline, findings have been inconsistent after adjustment for age, education, and other vascular risk factors (49), suggesting that their independent contributions may vary across study populations and analytical approaches. In our study, these null findings may also reflect exposure misclassification resulting from self-reported disease status, residual confounding by unmeasured factors such as disease duration, severity, treatment status, and genetic susceptibility, as well as survival bias inherent in community-based studies of older adults.

From a public health perspective, these findings suggest that community-based hypertension management programs may benefit from incorporating routine cognitive and mental health screening, particularly among high-risk groups such as the oldest-old, individuals with lower socioeconomic status, and those with depressive symptoms. Brief screening tools may facilitate early identification in primary care settings. Integrated management strategies addressing vascular, psychological, and socioeconomic factors may help support healthy cognitive aging in older adults with hypertension.

### 4.1 Strengths and limitations

This study has several strengths. It focuses on an understudied population: older adults with hypertension in a tropical region of China, providing novel, context-specific evidence. In addition, the community-based design and efficient sampling facilitated rapid, large-scale screening, improving the applicability of the findings to similar real-world community settings.

However, several limitations warrant consideration. First, the cross-sectional design precludes causal inference and determination of the temporal sequence of CI among older adults with hypertension. Therefore, the observed associations should be interpreted as exploratory rather than causal. Second, information regarding medical history and other questionnaire-based measures was obtained via self-report, which may be subject to recall bias and inaccuracies in reporting. Third, the absence of objective biomarkers, including directly measured blood pressure values and neuroimaging data, limited our ability to examine the biological mechanisms linking hypertension to CI. Fourth, cognitive function was assessed exclusively using the CMMS. Although the CMMS is a validated and widely used cognitive screening instrument in China, it is less sensitive than the Montreal Cognitive Assessment (MoCA) for detecting executive dysfunction and mild CI, particularly among individuals with hypertension. Consequently, some participants with subtle cognitive deficits may have been misclassified. Fifth, depressive symptoms were assessed using the PHQ-2 rather than the more comprehensive PHQ-9. Although the PHQ-2 has been validated as a screening instrument, it may underestimate depression severity or fail to identify subthreshold depressive symptoms. Finally, although formal interaction analyses were performed to evaluate effect modification, some subgroup analyses were based on relatively small sample sizes within certain strata, potentially reducing the precision and stability of subgroup-specific effect estimates. Therefore, these findings should be considered exploratory and interpreted cautiously until confirmed in larger prospective studies.

Future prospective cohort studies incorporating comprehensive neuropsychological assessments, more detailed depression measures, objective clinical indicators, neurobiological markers, and larger sample sizes are warranted to validate our findings and further elucidate the underlying mechanisms.

## 5 Conclusion

CI was common among community-dwelling older adults with hypertension in tropical China. Advanced age and depressive symptoms were associated with higher odds of CI, whereas higher educational attainment, moderate income, and higher BMI were associated with lower odds. A significant interaction between sex and marital status further suggests that social determinants of cognitive health may differ by sex. These findings provide context-specific evidence regarding factors associated with CI among older adults with hypertension and may help inform community-based strategies for cognitive and mental health screening. Future prospective studies are needed to confirm these associations and clarify their temporal relationships.

## Data Availability

The raw data supporting the conclusions of this article will be made available by the authors upon reasonable request. Requests for data access should be directed to the corresponding author, Jindong Ding Petersen, at dingjindong@muhn.edu.cn.
