# Supplementary material for: Cognitive impairment and associated factors among community-dwelling older adults with hypertension in tropical China
Source: Front Public Health. 2026 Aug 13;14:1900335. doi: 10.3389/fpubh.2026.1900335 (PMC13518148; doi:10.3389/fpubh.2026.1900335)
Supplement: Supplementary file 1 [file Table_1.docx]

***Supplementary Material***

**Table S1.** Univariate logistic regression analysis of factors associated with cognitive impairment among older adults with hypertension.

| Characteristic | *OR* (95% *CI*) | *p* |
| --- | --- | --- |
| Age, mean (SD), y | 1.05 (1.03 - 1.07) | <0.001 |
| Age groups, y |  |  |
| 60 - 69 | 1 [Reference] |  |
| 70 - 79 | 1.30 (0.90 - 1.86) | 0.159 |
| ≥80 | 2.78 (1.81 - 4.33) | <0.001 |
| Sex |  |  |
| Female | 1 [Reference] |  |
| Male | 0.64 (0.46 - 0.86) | 0.007 |
| Marital status |  |  |
| Married | 1 [Reference] |  |
| Other status | 1.70 (1.16 - 2.50) | 0.006 |
| Educational attainment, y |  |  |
| <1 | 1 [Reference] |  |
| 1 - 6 | 0.25 (0.14 - 0.46) | <0.001 |
| ≥7 | 0.23 (0.14 - 0.38) | <0.001 |
| Monthly income, yuan |  |  |
| Low <2000 | 1 [Reference] |  |
| Medium 2000 to <5000 | 0.46 (0.32 - 0.67) | <0.001 |
| High ≥5000 | 0.48 (0.31 - 0.75) | 0.001 |
| **Smoking status** |  |  |
| No | 1 [Reference] |  |
| Yes | 0.95 (0.57 - 1.58) | 0.849 |
| Alcohol consumption |  |  |
| No | 1 [Reference] |  |
| Yes | 0.62 (0.39 - 0.98) | 0.039 |
| Outdoor activity duration, h/day |  |  |
| <1 | 1 [Reference] |  |
| 1 - <2 | 1.02 (0.71 - 1.48) | 0.909 |
| 2 - <4 | 0.89 (0.55 - 1.44) | 0.645 |
| ≥4 | 1.51 (0.82 - 2.77) | 0.190 |
| BMI, mean (SD), kg/m^2^ | 0.93 (0.89 - 0.98) | 0.003 |
| Diabetes mellitus |  |  |
| No | 1 [Reference] |  |
| Yes | 1.16 (0.81 - 1.67) | 0.412 |
| Heart disease |  |  |
| No | 1 [Reference] |  |
| Yes | 0.72 (0.47 - 1.10) | 0.133 |
| Hyperlipidemia |  |  |
| No | 1 [Reference] |  |
| Yes | 0.81 (0.56 - 1.17) | 0.263 |
| Osteoarthritis |  |  |
| No | 1 [Reference] |  |
| Yes | 0.85 (0.59 - 1.22) | 0.377 |
| Depressive symptoms |  |  |
| No | 1 [Reference] |  |
| Yes | 2.10 (1.23 - 3.56) | 0.006 |
| **OR, odds ratio; 95%CI, 95%** confidence interval. | | |
