# Supplementary material for: Cognitive impairment and associated factors among community-dwelling older adults with hypertension in tropical China
Source: Front Public Health. 2026 Aug 13;14:1900335. doi: 10.3389/fpubh.2026.1900335 (PMC13518148; doi:10.3389/fpubh.2026.1900335)
Supplement: Supplementary file 2 [file Table_2.docx]

***Supplementary Material***

**Table S2.** Interaction analyses between demographic factors and cognitive impairment.

| Interaction term | Wald (df) | P for interaction |
| --- | --- | --- |
| Sex × Marital status | 5.895 | 0.015 |
| Sex × Educational attainment | 0.052 | 0.974 |
| df, degrees of freedom.  **Note:** Models were adjusted for age, sex, marital status, educational attainment, monthly income, alcohol consumption, BMI, and depressive symptoms. | | |
